# Influence of the Introduced Chitin Nanofibrils on Biomedical Properties of Chitosan-Based Materials

**DOI:** 10.3390/nano10050945

**Published:** 2020-05-15

**Authors:** Ekaterina N. Maevskaia, Anton S. Shabunin, Elena N. Dresvyanina, Irina P. Dobrovol’skaya, Vladimir E. Yudin, Moisey B. Paneyah, Andrey M. Fediuk, Petr L. Sushchinskii, Gerald P. Smirnov, Evgeniy V. Zinoviev, Pierfrancesco Morganti

**Affiliations:** 1 Department of Medical Physics, Peter the Great St. Petersburg Polytechnic University, Polytechnicheskaya Street 29, 195251 Saint Petersburg, Russia; anton-shab@yandex.ru (A.S.S.); elenadresvyanina@gmail.com (E.N.D.); zair2@mail.ru (I.P.D.); yudinve@gmail.com (V.E.Y.); 2 H.Turner National Medical Research Center for Children’s Orthopedics and Trauma Surgery, Parkovaya Street 64-68, 196603 Pushkin, Saint-Petersburg, Russia; 3 Institute of Textile and Fashion, Saint Petersburg State University of Industrial Technologies and Design, Bolshaya Morskaya Street 18, 191186 Saint Petersburg, Russia; tpnm@sutd.ru; 4 Laboratory of Mechanics of Polymers and Composites, Institute of Macromolecular Compounds, Bolshoy pr. V.O. 31, 199004 Saint Petersburg, Russia; 5 Laboratory of Experimental Surgery of Scientific Research Center, Saint Petersburg State Pediatrical Medical University, Litovskaya Street 2, 194100 Saint Petersburg, Russia; moisey031190@gmail.com (M.B.P.); andrej.fedyuk@gmail.com (A.M.F.); petersuchinsky@mail.ru (P.L.S.); evz@list.ru (E.V.Z.); 6 Saint-Petersburg I. I. Dzhanelidze Research Institute of Emergency Medicine, Budapeshtskaya Street 3, 192242 Saint Petersburg, Russia; 7 Department of Experimental Medicine, University of Campania Luigi Vanvitelli, via L. De Crecchio 7, 80138 Naples, Italy; pierfrancesco.morganti@mavicosmetics.it

**Keywords:** chitosan, chitin nanofibrils, hemostatic material, hemorrhage

## Abstract

Hemorrhage occurring during and after surgery still remains one of the biggest problems in medicine. Although a large number of hemostatic products have been created, there is no universal preparation; thus, the development of new materials is an urgent task. The aim of this research is to increase hemostatic properties of chitosan by introducing chitin nanofibrils (ChNF). The blood absorbance by ChNF-containing chitosan sponges and time-until-arrest of bleeding were studied. Non-woven materials containing 0.5% of ChNF and materials without chitin were obtained. The studies of ζ-potential showed that the material containing 0.5% ChNF had relatively a high positive charge, but efficiencies of both materials for hemorrhage arrest were comparable to those of commercial hemostatic products (Surgicel and TachoComb). To investigate the interaction between the materials and living organism, histological studies and optical microscopy studies were conducted after implantation of fibers. Despite bioinertness of fibers, implantation of non-woven materials led to formation of significant granulomas.

## 1. Introduction

Uncontrolled hemorrhage remains one of the most significant causes of mortality [1] in patients with traumas of various etiologies and medical conditions. Moreover, a subsequent blood loss leads to numerous possible complications and side effects in these patients, up to lethal hemorrhagic shock and multi-organ failure. Thereby, rapid and effective arrest of bleeding is an extremely important task. Nowadays, there is a huge variety of methods intended to reduce bleeding time and amount of blood loss, but the use of topical hemostatic materials remains the most popular and common way due to its simplicity, efficiency and ease of application. The ideal hemostatic material should be able to stop massive bleeding from large vessels and parenchymatous organs quickly and effectively to prevent repeated hemorrhage. Such properties as biocompatibility and ability for biodegradation are also quite important, as well as the absence of any negative effects in the adjacent tissues and major systems, ease of use and low cost [2,3]. Although numerous hemostatic materials are presented on the market, there is still no universal and ideal material; thus, development of new hemostatic products is an important task of medical materials science.

Hemostatic agents can be produced in various forms, such as films, sponges, hydrogels, granules, powders, fibers, as well as woven and non-woven materials based on these forms. Each form has its own limitations and advantages, which ensure high efficiency when a material is used in a certain specific case. Modern hemostatic materials are divided into several groups depending on the mechanism of action [4]: (1) physical agents, which provide occlusion of bleeding; (2) absorbing agents, whose main characteristic is swelling; (3) biologic agents that take part in biologic coagulation cascade; (4) synthetic agents (which, for example, glue the adjacent surfaces together). According to the literature, most polymers used to produce hemostatic materials are capable of realizing these mechanisms.

Thrombin and fibrin are widely used to create hemostatic agents, since they are directly involved in the process of blood clotting; therefore, they are highly effective. The same is true for microfibrillar collagen, which activates the intrinsic pathway of hemostasis reactions cascade. However, the use of human blood clotting factors significantly increases the risk of developing an immune response [5,6] as well as the cost of the product [7].

The materials which provide hemostasis due to blood absorbance and swelling are used much more frequently. This group of materials includes gelatin and starch, whose mechanisms of action are related to absorbance of the liquid blood phase; thus, the concentration of macromolecular blood clotting factors increases, and hemostasis is achieved [8]. Moreover, the material can expand and thereby limit the blood flow [9]. At the same time, the ability to expand is the main disadvantage of these materials, which restricts their use in the limited space [10].

Another example of the polymer commonly used in development of hemostatic materials is oxidized cellulose. Its mechanism of action is based on depolymerization with formation of cellulosic acid upon contact with wet environment. In this case, the local decrease in pH provokes lysis of red blood cells and release of hemoglobin, which turns into acidic hematin upon contact with cellulosic acid [11]. Decrease in the pH values contributes to the appearance of additional antimicrobial action [12]; however, in this case, possibility of inflammation and delayed wound healing increases [13]. In addition, this factor limits the joint use of hemostatic materials based on oxidized cellulose with other hemostatic agents [14].

Chitosan is one of the most promising polymers used in biomedicine, due to its biocompatibility and biodegradation properties as well as antibacterial effect and absence of cytotoxicity. As for hemostatic action, chitosan can serve as a promising base for hemostatic materials. In particular, such commercial products as Celox, HemCon and QuikClot demonstrate increased efficiency of bleeding arrest [15]. When these chitosan-based materials are used, hemostasis is achieved in a short period of time (from 53 s [16] to 6 min [17]), and this time depends directly on the nature of a damaged vessel and the species used in an experiment.

Various fillers are introduced to increase hemostatic properties of chitosan-based materials. For example, introduction of gelatin increases swelling degree of a material, and the use of oxidized cellulose reduces the period of resorption of a material in the body [18]. The additives can improve antibacterial properties of the product, reduce its cytotoxicity, promote faster wound healing and accelerate bleeding arrest, the latter being the most important parameter of a hemostatic material.

Since chitosan and chitin have similar natures, use of their combinations may enhance some properties necessary for biomedical applications. The materials containing chitin additives are widely used in tissue engineering, in development of drug delivery agents, wound dressings, anticancer preparations, antimicrobial agents [19]. There is information about the improved cell adhesion in the case of using low ratios of chitin nanoform [20] and enhancing of mechanical characteristics of materials [21]. The goal of this research was to obtain a chitosan-based material containing chitin nanofibrils (ChNF), evaluation of its hemostatic properties and study of interaction with living organism.

To obtain complete and reliable results, in vitro and in vivo studies should be used in combination, since each of these two methods has certain limitations. When in vitro methods are used, the experiment only approximately reproduces real conditions; all reactions that occur in the body cannot be taken into account; thus, reliability of this experiment is limited. Animal tests are associated with ethical difficulties, as well as with the error occurring due to individual characteristics; on the other hand, conditions of such experiments are significantly closer to the real conditions of clinical use [22].

The preliminary in vitro experiments involving chitosan-based composite materials were conducted in the previous works. It was shown [23] that upon the contact between blood plasma and composite chitosan-based ChNF-containing fibers, optical density of blood plasma decreases. This result is associated with sorption of hemoglobin by the fibers. The material containing 0.5% of ChNF demonstrated the least hemocompatibility due to the associated hemolysis processes. The behavior of composite fibers in wet environment was also investigated. This research was mostly dedicated to the in vivo studies of the behavior of composite materials. Chronic experiments provide an opportunity to study long-term interactions of a sample with living organism, tissue reactions, changes in cellular composition, thickness of the connective tissue capsule; it is also possible to reveal the presence or absence of the material’s bioresorption ability. It is believed that the optimal duration of the experiment is 14 days [22].

## 2. Materials and Methods

### 2.1. Materials

Chitosan, with molecular mass of 164 kDa and deacetylation degree of 92% (BiologHeppe, Landsberg, Germany) and chitin nanofibrils (ChNF) (SRL Mavi Sud, Aprilia, Italy) were used in our experiments. The length and diameter of ChNF were approximately 600 nm and 25 nm, respectively.

The following two commercial products with different mechanisms of action were used as control samples in hemostatic efficiency tests: Surgicel Nu-Knit consisting of oxidized cellulose (Ethicon, Somerville, NJ, USA) and TachoComb (Takeda Austria GmbH, Linz, Austria), which contains fibrinogen and thrombin as active substances.

### 2.2. Preparation of Composites

Chitin nanofibrils (ChNF) were preliminarily dispersed in water using an IL10-0.63 ultrasonic generator (Saint Petersburg, Russia) for 7 min at a frequency of 23.4 kHz. The mixture of chitosan and ChNF was dissolved in 2 wt% water solution of acetic acid. Concentrations of ChNF were 0.5 wt%, 5 wt% and 50 wt% with respect to chitosan amounts; total concentrations of polymers in the solution were 3 wt% (for sponges) and 4 wt% (for fibers) [24]. The prepared solutions were filtered and deaerated at a pressure of 0.1 atm.

The sponges were obtained by lyophilization. The solutions were preliminary frozen at −20 °C, then lyophilized at −2 °C and at a pressure of 1.6 Pa with the aid of a LABCONCO Triad Freeze Dry System (Labconco Corporation, Kansas City, MO, USA). In order to transform the sponges into the insoluble basic form, they were treated with 10% water solution of NaOH and then washed with distilled water.

The fibers were obtained by wet spinning [24]. The solutions were fed through a spinneret die into coagulation bath (10% water solution of NaOH and ethanol, 1:1), then the obtained fibers were washed with distilled water and dried at 40 °C. In order to produce monofilaments, the die with the hole diameter of 0.6 mm was used; polyfilaments were spun using a die with 100 holes, diameter of each hole was 100 μm. The feed rate during monofilament preparation was 0.2 mm/min; in the case of polyfilaments, feed rate was 0.3 mm/min. During spinning, oriented fibers were obtained due to 50% drawing. The non-woven material was obtained by needle-punching from polyfilament fibers.

### 2.3. Measurements of Blood Absorbance by Chitosan Sponges

The sponges were cut into pieces of the same area (10 × 10 mm^2^) and different thicknesses (3 and 5 mm). After pre-weighing (W1), the sponges were brought into contact with rat blood for 1 min and weighed again (W2). The absorbance ability of sponges (W) was calculated as a difference between the weights: W = W2 − W1. The obtained parameters were compared taking into account sponge thicknesses and ChNF concentrations.

### 2.4. Measurements of Ζ-Potential

The measurements of ζ-potential of non-woven materials were carried out using a SurPASS 3 instrument (Anton Paar, Graz, Austria) according to the method described in [25]. Briefly, the samples (size: 10 × 20 mm^2^) were mounted on sample holders in such a fashion that the gap between them was approximately 100 μm. The pressure was varied from 600 mbar to 200 mbar; the temperature was 23 °C. Solution of KCl (0.001 mol/L) was used as the background electrolyte; the pH value was varied from 5 to 9 by addition of 0.05 mol/L KOH.

### 2.5. In Vivo Experiments 

The use of experimental animals in the studies of the obtained materials was permitted by the Local Ethics Committee of Saint-Petersburg State Pediatric Medical University (Saint-Petersburg, Russia). 

All manipulations were performed under full anesthesia (mixture of tiletamine hydrochloride and zolazepam hydrochloride) in strict compliance with the European Convention for the Protection of Vertebrate Animals used for Experimental and other Scientific Purposes (ETS 123). Euthanasia was performed in strict compliance with the Recommendations for Euthanasia of Experimental Animals of European Commission.

Sixty-five animals were used in the studies (Wistar Kyoto male rats, body weight 230–250 g). The animals were divided into 3 groups for different experiments.

#### 2.5.1. Experiments Related to Arrest of Arterial and Vein Bleeding

The first group of 54 experimental animals was used in the experiments involving stop of bleedings from femoral artery and vein. The mixture of tiletamine hydrochloride and zolazepam hydrochloride was used for anesthesia (Zoletil 100, Vibrac, France). The dosage was calculated individually for each animal (15 mg per 1 kg of the animal’s mass). Intramuscular injections of the anesthetic mixture were administered.

To simulate femoral bleeding under conditions of complete anesthesia, the inner side of the animal thigh was shaved, and the animal was fixed to the operating table with ligatures. An incision 1 cm long was made on the inner side of the thigh, and a frame retractor was introduced into the incision. The fascia was dissected using a scalpel and microsurgical scissors, access to the neurovascular bundle was ensured, then the femoral artery (vein) was isolated. When the selected blood vessel was intersected, the studied material (7 × 7 × 4 mm^3^) was applied to the damaged area. The time until the complete arrest of bleeding was measured.

Eight samples were investigated in 2 phases. The first phase included tests of chitosan sponges without ChNF and the sponges containing 0.5 wt%, 5 wt% and 50 wt% of ChNF as materials for bleeding arrest. At the second stage, pure chitosan non-woven materials and chitosan-based non-woven materials containing 0.5% of ChNF were compared with commercial hemostatic materials and the control group. The materials that demonstrated the best properties in the above experiments were selected for further studies. The reference groups included commercial materials (TachoComb and Surgicel) of similar sizes. In the control experiments, hemostatic agents were not used.

#### 2.5.2. Studies of Resorption and Implantation

To investigate the potential tissue response to the studied materials and to make preliminary assessments of applicability, implantation tests were performed in the second group of animals. All investigated materials were introduced into subfascial plane in the projection of both m. latissimus dorsi of 3 intact rats for a period of 14 days. The distance between the samples was at least 1 cm to avoid cross-exposure. In 14 days after the beginning of the experiment, biopsy samples were taken.

Chitin–chitosan fibers were introduced into subfascial plane of m. latissimus dorsi of 8 intact rats for 14 and 91 days. The introduced chitosan and chitin–chitosan (0.5%) fibers were used as a basis for preparation of the non-woven materials mentioned above. The experiments involving long-term implantation of singular elements allowed us to assess the consequences of destruction of materials in the wound when these materials are removed after use.

After processing the skin on the back in the region of m. latissimus dorsi, a 1 cm incision was made up to the fascia. A frame retractor was introduced into the section, and the edges of the section were fixed to facilitate the ongoing manipulations. The fascia was incised and moved apart with the help of pointed four-tooth hooks. The test samples were placed under the fascia. After a specimen was placed into the desired area, the fascia was sutured with the Vicryl 4-0 suture. The distance between the suturing area and the area containing the specimen was at least 1 cm to exclude the influence of suture material. The skin incision was sutured using the Vicryl 2-0 suture.

### 2.6. Histological Studies

Tissue samples were fixed in 10% solution of neutral formalin in phosphate buffer (pH = 7.4) for 24 h, then treated with ethanol solutions of increasing concentrations (10%, 30%, 50%, 80% and 99%) and embedded in paraffin. Paraffin sections (5 μm thick) were dyed with hematoxylin and eosin (Bio-Optica, Milan, Italy). Microscopic analysis and registration of images were performed with the use of a Leica DM750 light microscope (Wetzlar, Germany).

### 2.7. Statistical Processing of Data

Statistical analysis of the results was carried out using the Origin software (OriginLab Corporation, USA) and the R software (R Foundation for Statistical Computing, Vienna, Austria). The Kruskal–Wallis test and Mann–Whitney test were used to compare the independent samples. The differences were considered statistically significant when the p-value was less than 0.05.

## 3. Results

### 3.1. Preparation of Non-Woven Materials

**Figure 1:**
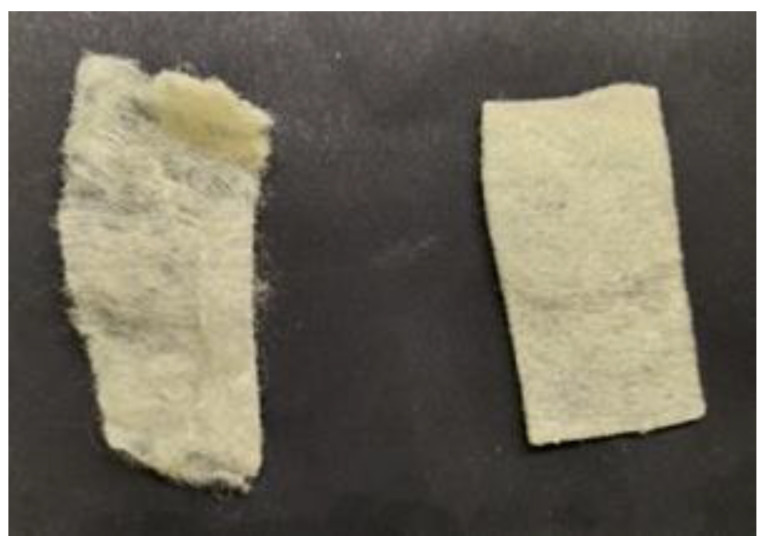
Non-woven material without chitin nanofibrils (ChNF) (left) and the material containing 0.5% ChNF (right).

Addition of ChNF to chitosan solutions facilitates wet spinning of fibers, which, in turn, leads to enhancement of their mechanical properties [24] and improvement of the structure of the resulting filaments and the non-woven material produced on their basis (Figure 1). This phenomenon can be explained by the fact that introducing a low amount of ChNF (0.3–0.5%) into the chitosan matrix ensures additional orientation of the chitosan macromolecules on their surface [24]. Orientation of chitosan macromolecules on the surface of ChNF was also confirmed [24] by energy minimization and molecular dynamics simulation of the systems containing one chitosan molecule on the surface of chitin nanocrystallite. Mutual ordering of the components of the mixture contributes to the formation of hydrogen bonds between them, this resulting in the formation of a more stable and energetically favorable structure.

### 3.2. Measurement of Blood Absorbance by Chitosan Sponges

**Figure 2:**
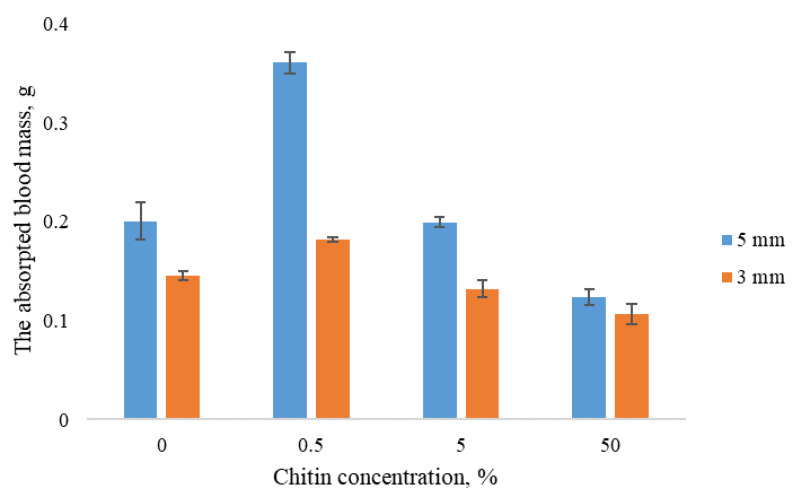
Blood absorbance by sponges vs. ChNF content in samples.

For all samples, an increase in sample thickness led to higher blood absorption (Figure 2). Regardless of the size, the highest value of blood absorption was shown by the sponges containing 0.5% ChNF; this effect becomes more pronounced with increasing sample size.

### 3.3. Arrest of Bleeding

**Figure 3:**
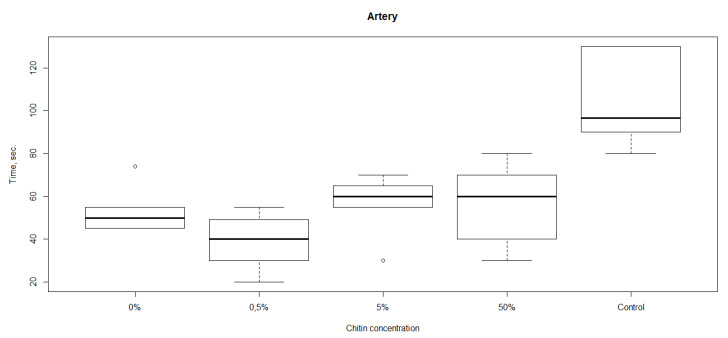
Rates of bleeding arrest (time until cessation of bleeding) in the experiments with chitosan sponges containing different amounts of ChNF.

The results of studies of hemostatic properties of the chitosan sponges containing different amounts of ChNF are shown in Figure 3. It is seen that all materials demonstrate hemostatic properties, i.e., the bleeding from femoral artery is arrested faster than that in the case when the materials were not applied (the control group). The fastest bleeding arrest was observed in the experiment involving the material with 0.5% ChNF. Therefore, this concentration was used in preparation of non-woven materials.

**Figure 4:**
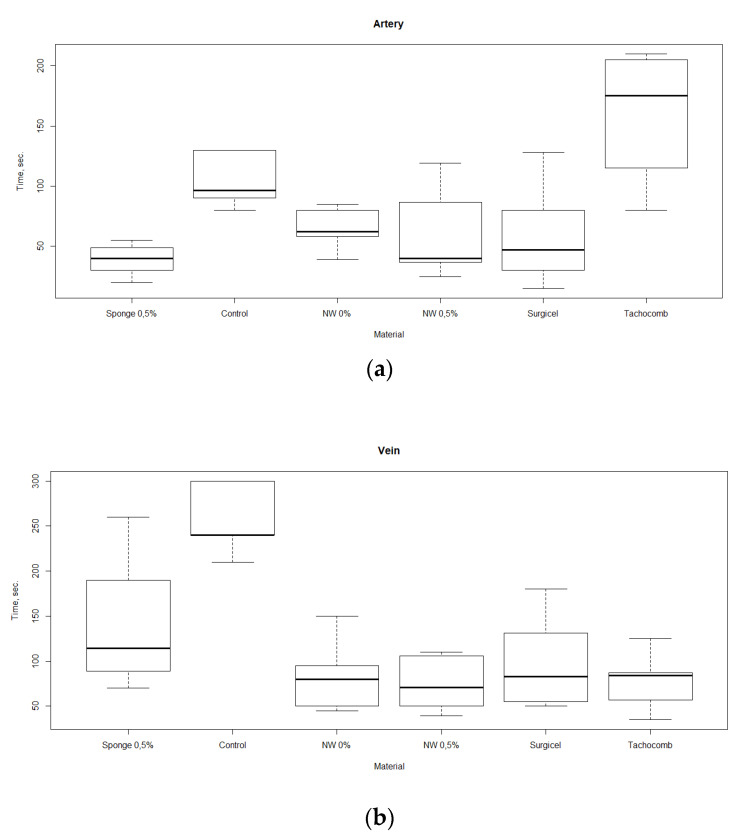
Rates of bleeding arrest (time until cessation of bleeding) in the experiments with different materials, in which femoral (**a**) and vein (**b**) artery hemorrhages were modeled.

The results of studies of bleeding arrest by non-woven materials are shown in Figure 4. It can be noted that for venous bleeding, both non-woven materials (the “NW” group in the picture) and the commercial samples showed higher effectiveness in comparison with that of a sponge. At the same time, in the case of arterial bleeding, only non-woven material containing ChNF demonstrated the hemostatic properties comparable to those of the sponge material with the same ChNF concentration. In both cases, the non-woven material showed the efficiency comparable with those of commercial products, and arterial bleeding stopped even faster than in the experiments with TachoComb.

### 3.4. Measurements of Ζ-Potential

**Figure 5:**
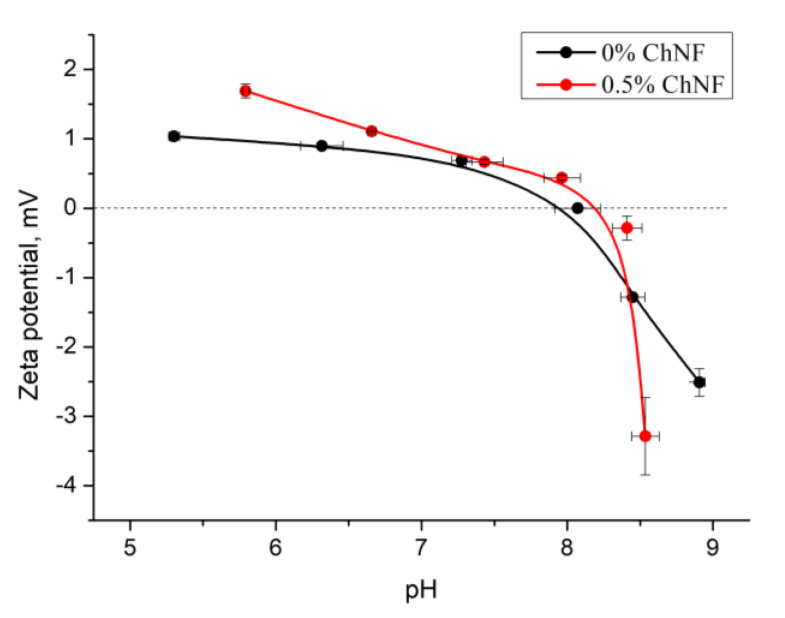
The pH dependences of ζ-potential of non-woven materials.

Hemostatic properties of the obtained materials described above can be explained by the value of the charge present on the composite surface. The pH dependences of the ζ-potential of the non-woven materials based on chitosan fibers and ChNF-containing composite fibers are shown in Figure 5. It is seen that both fibers have positive surface charges. Introducing ChNF in small amounts (0.5 wt%) leads to an increase in the surface potential of the composite fiber.

It is known [26] that chitosan possesses polyelectrolytic properties. In solutions of weak acids, amino group (NH_2_) is transformed into positively charged NH_3_^+^ group; hydroxyl and amino groups of chitosan form internal and intermolecular hydrogen bonds. It can be assumed that regular arrangement of chitosan macromolecules in a composite fiber (in particular, their mutual orientation), as well as their orientation relative to ChNF, will have a certain influence on the number of hydrogen bonds and, as a result, on the value of surface charge of the fiber. If macromolecules have stretched conformation, probability of formation of chitosan intramolecular bonds will be reduced, thereby increasing the free surface charge of the fiber.

It has been shown [24] that introducing ChNF into chitosan solution facilitates orientation of chitosan macromolecules on the surface of nanoparticles. In addition to the hydrogen bonds described above, chitosan amino groups can interact with carbonyl –C = O– groups of chitin. As indicated by the data shown in Figure 5, introduction of a small amount of ChNF (0.5 wt%) increases the value of the ζ-potential. A sharp decrease in the potential occurs when pH of the medium reaches the isoelectric point.

### 3.5. Studies of Degradation of Fibers in Organism

**Figure 6:**
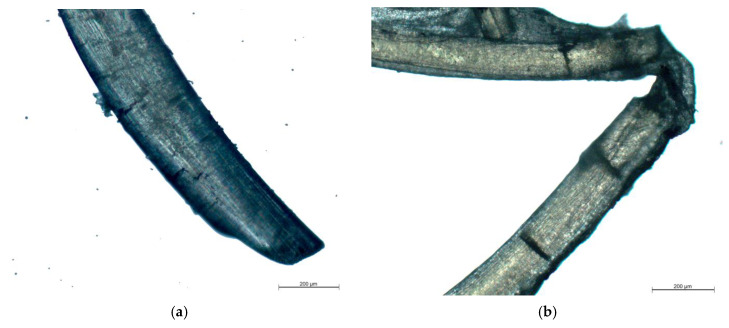
Images of the chitosan fiber (0.5% ChNF) located under m. latissimus dorsi that underwent bioresorption for (**a**) 14 days and (**b**) 91 days.

In 2 weeks after introducing the fibers into organism, weak signs of fiber fragmentation are observed (Figure 6). Small cracks appear on fiber surface. In 91 days after implantation, a dense connective tissue capsule appears (which is confirmed by histological studies). Despite partial fragmentation, the resulting pieces of material are held together by the capsule. Resorption of chitosan fibers may be associated with mechanical loads appearing during the movements of rats due to contact between fibers and muscles.

### 3.6. Histological Studies

#### 3.6.1. Histological Studies of Tissues around Implanted Fibers

**Figure 7:**
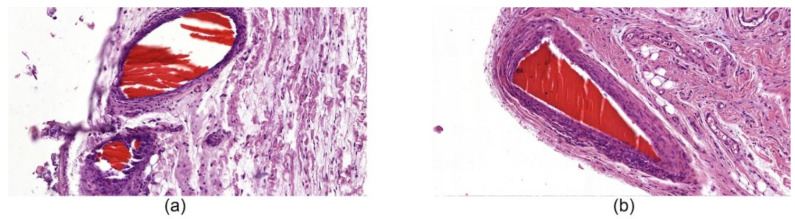
Histological microphotographs of biopsy samples of [chitosan + 0.5% ChNF] fibers, magnification 50×. (**a**) subfascial implantation, day 14; (**b**) subfascial implantation, day 91.

In the biopsy samples of the ChNF-chitosan filaments (0.5% of chitin) used in subfascial implantation, we observed a circle of coarse, fibrous connective tissue containing numerous fibroblasts with large bright oval-shaped nuclei. It was formed around the material in 14 days after beginning of the experiment (Figure 7a). In addition, focal lymphomacrophagous infiltration with an admixture of neutrophilic leukocytes (1–3 in the field of view, magnification 400×) and foreign body giant cells (1–2 in the field of view, magnification 400×) were found. On the 91st day of observation (Figure 7b), no signs of fiber resorption were observed. A narrow rim of scar tissue with small foci of lymphomacrophagous infiltration and an admixture of single neutrophilic leukocytes were present in the field of view (magnification 400×) around the fibers.

#### 3.6.2. Histological Studies of Tissues around Implanted Hemostatic Materials

**Figure 8:**
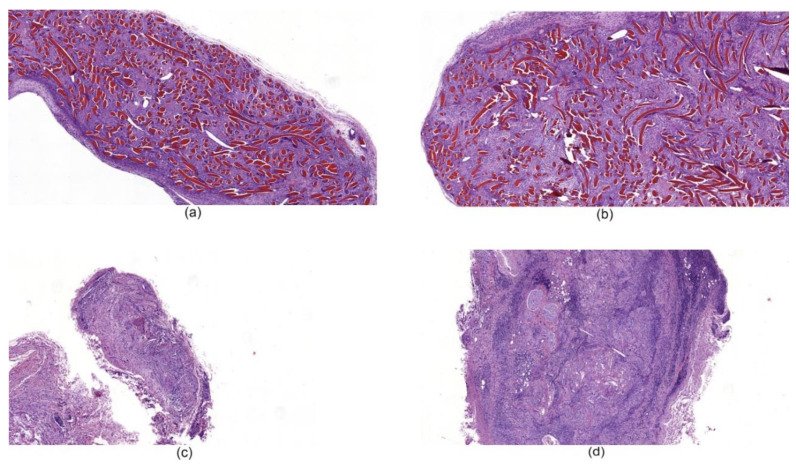
Histological microphotographs of biopsy samples of hemostatic materials taken on the 14th day of the experiment, magnification 50×. (**a**) non-woven chitosan material; (**b**) [chitosan + 0.5% ChNF] non-woven material; (**c**) “Surgicel” hemostatic material; (**d**) “TachoComb” hemostatic material.

**Figure 9:**
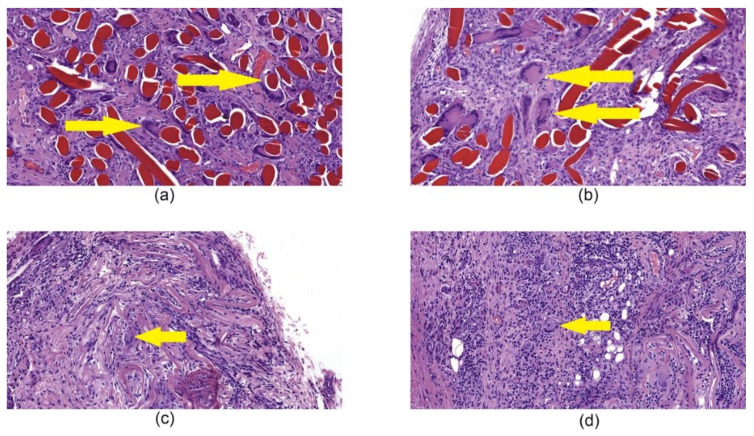
Histological microphotographs of biopsy samples of tissues with implanted hemostatic materials taken on the 14th day after beginning of the experiment, magnification 200×. (**a**) chitosan non-woven material, the arrow points to giant cells in granuloma; (**b**) [chitosan + 0.5% ChNF] non-woven material, the arrow points to giant cells in granuloma; (**c**) “Surgicel” hemostatic material, the arrow points to giant cells; (**d**) “TachoComb” hemostatic material, the arrow points to giant cells between infiltration.

Histological examination of tissues with implanted chitosan hemostatic non-woven materials performed on the 14th day of observation revealed a large macrophage granuloma surrounding brightly eosinophilically stained chitosan fibers (Figure 8a). In addition, numerous foreign body giant cells were detected in the granuloma (Figure 9a). In the group of animals with implanted chitosan hemostatic sponges containing 0.5% of ChNF, a large granuloma with numerous foreign body giant cells and a small number of infiltrated neutrophilic leukocytes were detected among brightly stained eosinophilic filaments (Figure 8b). The group of samples obtained from the animals with implanted “Surgicel” hemostatic sponge shows a small sclerosis focus with small focal lymphomacrophagic infiltration and low amount of foreign body giant multinuclear cells in the field of view (Figure 8c).

## 4. Discussion

The best results in the blood sorption experiments and the best hemostatic properties were revealed for the chitosan sponges containing 0.5% of ChNF. Therefore, the non-woven material containing this amount of ChNF was prepared and used in the experiments involving arrest of venous and arterial bleeding. Efficiency of this material was comparable to those of commercial hemostatic agents (Surgicel and TachoComb). TachoComb demonstrated somewhat deteriorated properties; this result may be explained by the fact that it contains human fibrinogen and thrombin, while the experiments were conducted in rats. It also should be noted that the Surgicel application instruction forbids its use in the cases of massive artery hemorrhage; thus, chitosan-based materials can be used as substitutes.

In order to reveal possible causes of different hemostatic effect of non-woven materials, the measurement of ζ-potential was carried. It was shown that addition of 0.5% of ChNF leads to the increment of positive charge at pH value from 5 to 8; after the isoelectric point is achieved, sharp decline occurs. It can be assumed that this phenomenon is related to higher ordering of the surface structure of composite fibers, more regular arrangement of positively charged groups. In [24], it was shown that introduction of 0.5 wt% of ChNF increases the orientation of chitosan macromolecules due to adhesion of chitosan macromolecules on the surface of ChNF. Model calculations performed by molecular dynamics have shown that the most energy-efficient location of chitosan macromolecules is along the ChNF axes. The orientation of chitosan macromolecules, including their localization on fiber surface, may lead to regular arrangement of positive charges and thus may minimize their interaction with other groups of chitosan and chitin macromolecules (primarily carboxylic and carbonyl fragments). Moreover, the sharp drop in the ζ-potential at the isoelectric point may indicate higher ordering of the charged groups of composite fibers and, as a result, narrower distribution of the charge value.

The existence of higher positive charge on the surface of non-woven material containing 0.5% of ChNF can explain better interaction of this material with blood cells (which are negatively charged). Moreover, finer structure of the composite non-woven material may be related to increased plasma sorption. To study the subsequent interaction of materials with organism, the samples were placed under the fascia of m. latissimus dorsi for 14 days.

According to the results of histological examination, the implanted chitosan filaments containing 0.5% of ChNF demonstrate bioinertness. An extremely low number of foreign body giant cells and leukocyte infiltration were observed on the 91st day in the case of subfascial implantation. In addition, a connective tissue capsule was formed around the fiber. Taking these results into account, we studied implantation of non-woven materials based on fibers of similar composition. Nevertheless, implantation of such material led to the formation of a significant granuloma, while low degree of resorption of the tested material was observed. At the same time, the commercial analogs (Surgicel and TachoComb) demonstrated high degrees of resorption on the 14th day of the experiment with the formation of separate foci of sclerosis.

The long-term study of behavior of fibers in organism showed that biodegradation signs appear in 3 months after implantation into abdomen and m. latissimus dorsi. This result may be connected to the mechanical stress during the rats’ movement and to chitosan ability for bioresorption.

## 5. Conclusions

In this work, we studied the influence of ChNF additives present in chitosan-based materials on hemostatic properties of samples. The sponges containing 0.5% of ChNF demonstrated the best results in the experiments involving blood absorbance in vitro, and the highest rate of arterial bleeding arrest in vivo. Therefore, this concentration was chosen for preparation of the non-woven material. The quality of ChNF-containing material was higher than that of pure chitosan samples, but efficiencies of both materials were comparable with those of commercial hemostatic agents Surgicel and TachoComb. The studies of ζ-potential indicate better interaction between the chitin-containing material and blood cells due to higher surface positive charge of this material. This high surface charge appears due to orientation of chitosan macromolecules along the ChNF. To study the long-term interaction of the materials with organism, ChNF-containing chitosan fibers were implanted into rats. The histological studies demonstrated bioinertness of the fibers, and light microscopy studies showed the signs of biodegradation in 91 days after implantation due to their fragmentation. Meanwhile, histological studies of non-woven materials indicated formation of granulomas with foreign body giant cells; therefore, the detailed further research of the process is required.
